# The miR172c-NNC1 module modulates root plastic development in response to salt in soybean

**DOI:** 10.1186/s12870-017-1161-9

**Published:** 2017-12-01

**Authors:** Zulfiqar Ali Sahito, Lixiang Wang, Zhengxi Sun, Qiqi Yan, Xingke Zhang, Qiong Jiang, Ihteram Ullah, Yiping Tong, Xia Li

**Affiliations:** 1 State Key Laboratory of Agricultural Microbiology, College of Plant Science and Technology, Huazhong Agricultural University, Wuhan, 430070 People’s Republic of China; 2 Institute of Genetics and Developmental Biology, Chinese Academy of Sciences, 286 Huaizhong Road, Shijiazhuang, Hebei 050021 People’s Republic of China; 3 University of Chinese Academy of Sciences, Beijing, 100049 People’s Republic of China; 4 State Key Laboratory of Plant Cell and Chromosome Engineering, Institute of Genetic and Developmental Biology, Chinese Academy of Sciences, Beijing, 100101 China

**Keywords:** miR172c, Root development, Salt stress, Soybean

## Abstract

**Background:**

Plant roots are highly plastic to high salinity. However, the molecular mechanism by which root developmental plasticity is regulated remains largely unknown. Previously we reported that miR172c-*NNC1* module plays a key role in soybean-rhizobial symbiosis. The fact that the miR172c promoter contains several stress-related *cis* elements indicates that miR172c may have a role in root response to abiotic stress.

**Results:**

Here we showed that miR172c is greatly induced by salt stress in soybean. Overexpression of miR172c and knockdown of miR172c activity resulted in substantially increased and reduced root sensitivity to salt stress, respectively. Furthermore, we show that the target gene *NNC1* (*Nodule Number Control 1*) of miR172c was downregulated by salt stress. The transgenic roots overexpressing or knocking down *NNC1* expression also exhibited the altered root sensitivity to salt stress.

**Conclusion:**

The study reveals the crucial role of miR172c*-NNC1* module in root stress tolerance to salt stress in soybean.

**Electronic supplementary material:**

The online version of this article (10.1186/s12870-017-1161-9) contains supplementary material, which is available to authorized users.

## Background

Due to its high seed protein and oil contents, soybean is considered as an important crop globally, both for human food and animal feed. Soybean is also well-known for its different root system with specialized lateral root organs, root nodules, which can fix atmospheric N_2_ into the plant usable NH4^+^ [1]. Like other important crops, soybean is a typical glycophyte that is sensitive to high contents of salt in root zone. With increasing extensive application of fertilizers and global warming, salinity has become a major constraint in soybean production globally [2]. Understanding soybean response to salt stress and, simultaneously, its genetic improvement for salt tolerance is a challenge for plant scientists.

In the past decades, extensive efforts have been made to elucidate the molecular mechanisms underlying salt tolerance in soybean. Using a combinatorial approach, several genes that mediate soybean plant responses to salt stress have been identified. These soybean genes are involved in various biological processes [3]. For example, *GmSALT3* (Salt tolerance-associated gene on chromosome 3) cloned by map-based strategy encodes an ion homeostasis cation/H^+^ exchanger and positively regulates salt tolerance in soybean [4]. But the majority of salt tolerance-related genes that mediate transcription activation or repression of the downstream genes are mainly transcription factor genes, such as *ERF*, *DREB*, *WRKY, MYB*, *bZIP* and *GmNAC* family genes [5–10]. Recent research showed that these transcription factors may coordinately regulate plant stress tolerance through directly targeting other transcription factors. For example, GmWRKY27 physically interacts with GmMYB172 to repress the expression of *GmNAC29* that modulates plant tolerance to salt and drought stresses in soybean [9]. These results highlight the pivotal roles of the transcription factor-mediated reprogramming in soybean adaptation to salt stress. Despite the great progresses, the upstream regulators of these salt stress-related transcription factors in soybean remains elusive.

Genome-wide analyses of microRNAs (miRNAs) in various organisms have revealed that nearly 50% of the target genes of miRNAs are the genes that associate with transcription regulation, including various transcription factors [11]. These findings implicate that miRNAs function as the major upstream regulators of the transcription factors that modulate various biological processes. miRNAs are small non-coding RNAs that negatively regulate their target genes through cleaving the target mRNAs or reducing the activity of the target genes at posttranslational level [12]. With the aim to uncover the miRNAs-mediated regulatory network and identify the miRNAs that mediate plant responses to salt, many global analyses of miRNAome in response to salt stress have been performed in various plant species [13–18]. The extensive datasets generated from these analyses have confirmed that miRNAomes are highly plastic in response to salt stress, and miRNAs are key regulators of plant tolerance to salt stress. These miRNAs including miR156, miR158, miR165, miR167, miR168, miR169, miR171, miR393, miR394 and miR396 were responsive to salt stress and may fine-tune plant stress responses at multiple levels through targeting the genes with different functions including large sets of genes that encode various transcription factors [15, 19, 20]. Further functional analyses of several miRNAs, such as miR169 and miR393, demonstrate that miRNAs indeed play a key role in plant tolerance to salt stress [21, 22].

miR172 has been widely recognized for their role in the regulation of flowering time and developmental phase transition in Arabidopsis and other plants [23, 24]. Recently, our results together with others have convincingly shown that miR172 is a key regulator of nodulation in legumes [25–28]. Interestingly, miR172 family members were also in the lists of the salt-responsive miRNAs [19]. The compelling evidence points to the divergent roles of miR172 in plant development and stress response. Intriguingly, the fact that the soybean miR172c promoter contains several ABRE *cis* elements prompted us to investigate whether miR172c mediates soybean plant response to salt stress. In this study, we found that miR172c was highly induced by salt stress in soybean roots, and demonstrated that miR172c is a positive regulator in root plastic development in response to salt stress in soybean. We also revealed that miR172c and its target gene NNC1 (Nodule Number Control 1), an AP2 transcription factor family gene, is an important module in soybean salt tolerance. Our results provided novel insights into the miRNA-based regulatory mechanism of developmental plasticity and plant adaptation to salt stress.

## Results

### Soybean miR172c is induced by salt stress

**Fig. 1:**
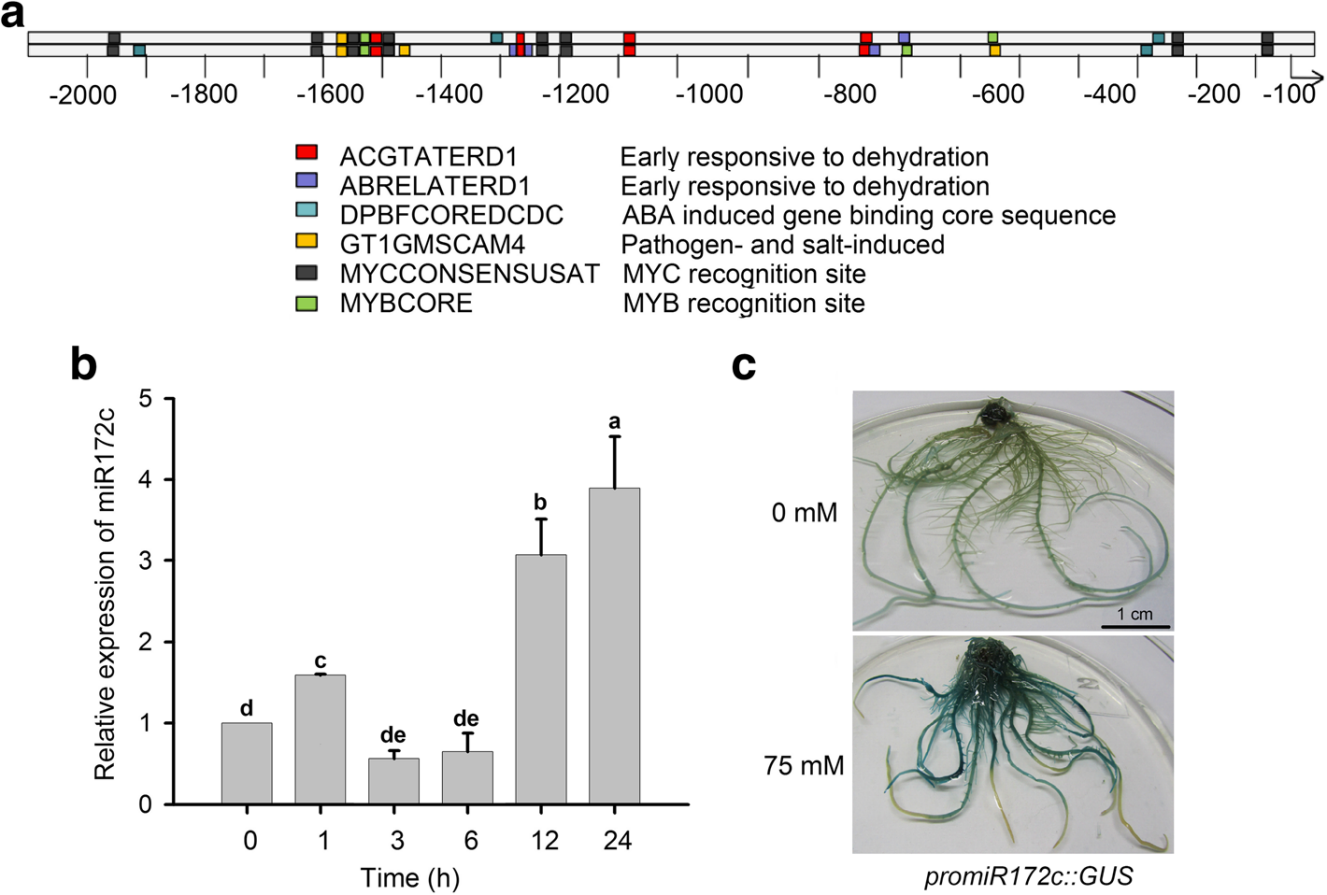
Analysis of the miR172c promoter and miR172c expression. **a** Computational analysis of the regulatory *cis* elements of the miR172c promoter. The promoter sequence (2000 bp) of upstream of pre-miR172c (www.phytozome.net/) was chosen for multiple *cis*-element analysis using an online software at http://www.dna.affrc.go.jp/place/. **b** qRT-PCR analysis of expression of miR172c in roots. Seven day-old seedlings were treated with 75 mM NaCl, and roots were harvested at 0, 1, 3, 6, 12, and 24 h after treatment. miR1515a was used to normalize transcript abundance. Data are mean ± SD from three biological repeats. Letters indicate significant differences from the empty vector controls according to the Student’s Newman-Kuels test (*P* < 0.05). **c** Histochemical analysis of the expression of miR172c. GUS staining was performed using the transgenic roots expression *promiR172c::GUS* treated with or without 75 mM NaCl for 24 h. Two weeks-old composite plants were used to conduct the experiments. Bars in (**c**) =1 cm

Analysis of the miR172c promoter reveals manifold *cis*-regulatory elements related to early responses to dehydration and abscisic acid (ABA), including ACGTATERD1, ABRELATERD1, DPBFCOREDCDC, MYB2AT and MYBCORE (Fig. 1a). This observation made us curious about whether miR172c also perform a role in soybean plant response to osmotic stress. To test the possibility, we first performed a qRT-PCR analysis to examine whether miR172c is responsive to salt stress. When the soybean seedlings were subjected to salt stress on B5 medium containing 75 mM NaCl, miR172c was rapidly induced in roots and the expression levels of miR172c were continuously elevated during the prolonged exposure treatment (Fig. 1b). The result clearly showed that miR172c was indeed salt responsive in soybean, pointing a role of miR172c in soybean root response to salt stress.

Previous results have shown the importance of cell-type specific response to salt stress [29]. To determine whether miR172c mediated cell/tissue specific response of soybean seedlings to salt stress, we generated the composite transgenic plants expressing *miR172cpro:GUS* gene using a hairy root transformation system. As shown in Fig. 1c, the miR172c showed weak expression in soybean transgenic roots with the higher level of expression in older roots close to the junction sites between stems and roots under normal conditions. Upon salt stress, miR172c was highly induced in the treated soybean roots close to the shoot-root junctions, while the expression of miR172c remained unchanged in the root tip regions (Fig. 1c). These results confirmed our hypothesis that miR172c may be a key regulator in plastic development of root system under salt stress.

### Overexpression of miR172c promotes hairy root development and increases root tolerance to salt stress

To test whether miR172c functionally mediates plant response to salt stress in soybean, we generated the composite transgenic plants overexpressing the miR172c under CaMV35S promoter. After co-cultivation, the explants were transferred to growth containers containing MS rooting medium for one week, and the rooted composite plants were then transferred to vermiculite treated without or with 75 mM NaCl for 15 days. During hairy root initiation stage, we found that overexpression of miR172c promoted hairy root system under control conditions (Additional file 1a, 1b). The lengths of miR172c overexpression roots were significantly longer than that of the vector controls, and the number of lateral roots per transgenic root overexpressing miR172c was also substantially increased compared to the control. It is apparent that overexpression of miR172c stimulates initiation of hairy roots.

**Fig. 2:**
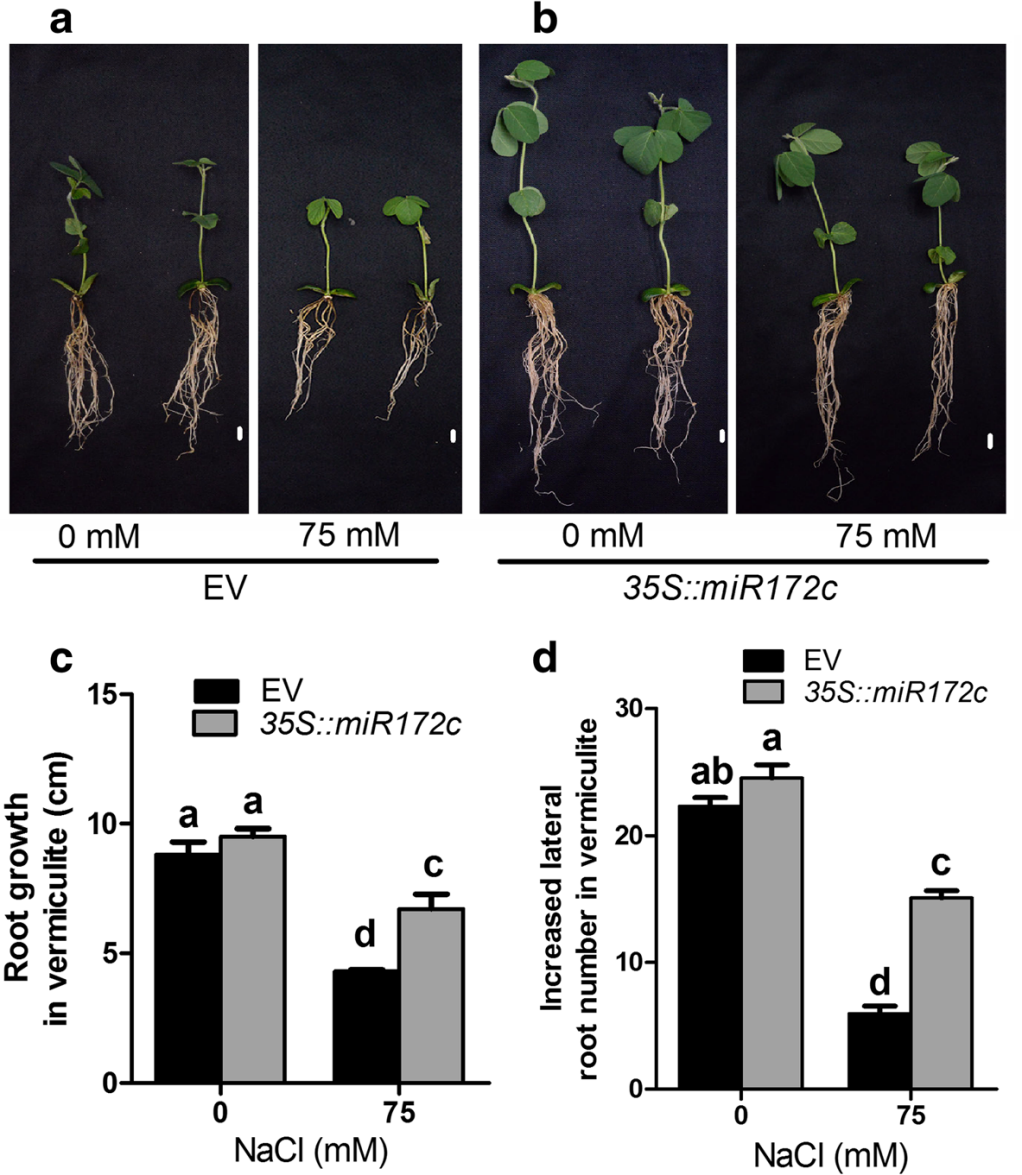
Overexpression of miR172c increases salt tolerance of soybean roots. **a** and **b** Phenotype analysis of the composite plants and individual hairy root overexpressing *35S::miR172c* and the empty vector. Seven day-old composite plants grown in B5 medium were transplanted to vermiculite and treated without (**a**) and with 75 mM NaCl (**b**). Bars = 1 cm. **c** and **d** Increases in root system lengths (**c**) and lateral numbers after transplanting the composite plants expressing the empty vector and 35S:miR172c to soil Letters indicate significant differences from the empty vector controls according to the Student’s Newman-Kuels test (*P* < 0.05).

Next, we evaluated root systems of the miR172c overexpressers and the vector controls at 15 days after salt treatment, and the increases in root system lengths and lateral root numbers grown in vermiculite were measured. Interestingly, in the absence of salt stress, the overall root system growth of the miR172c overexpressors in soil including root system lengths and lateral development were not significantly different from that of the vector controls, although shoots of miR172c overexpressors exhibited much better growth compared with that of the controls (Fig. 2a, b). However, the root system of the composite plants overexpressing miR172c showed dramatically enhanced salt tolerance compared to that of the vector control at 15 days after treated with salt (Fig. 2a, b). The root lengths of miR172c overexpressorss were much longer than that of the vector controls, and in particular the number of lateral roots per transgenic root overexpressing miR172c was substantially increased compared to the control (Fig. 2c, d). The data indicated that miR172c positively regulates root plastic development and salt tolerance in soybean.

### Reduction of miR172c activity causes delayed root development and increased root sensitivity to salt stress

**Fig. 3:**
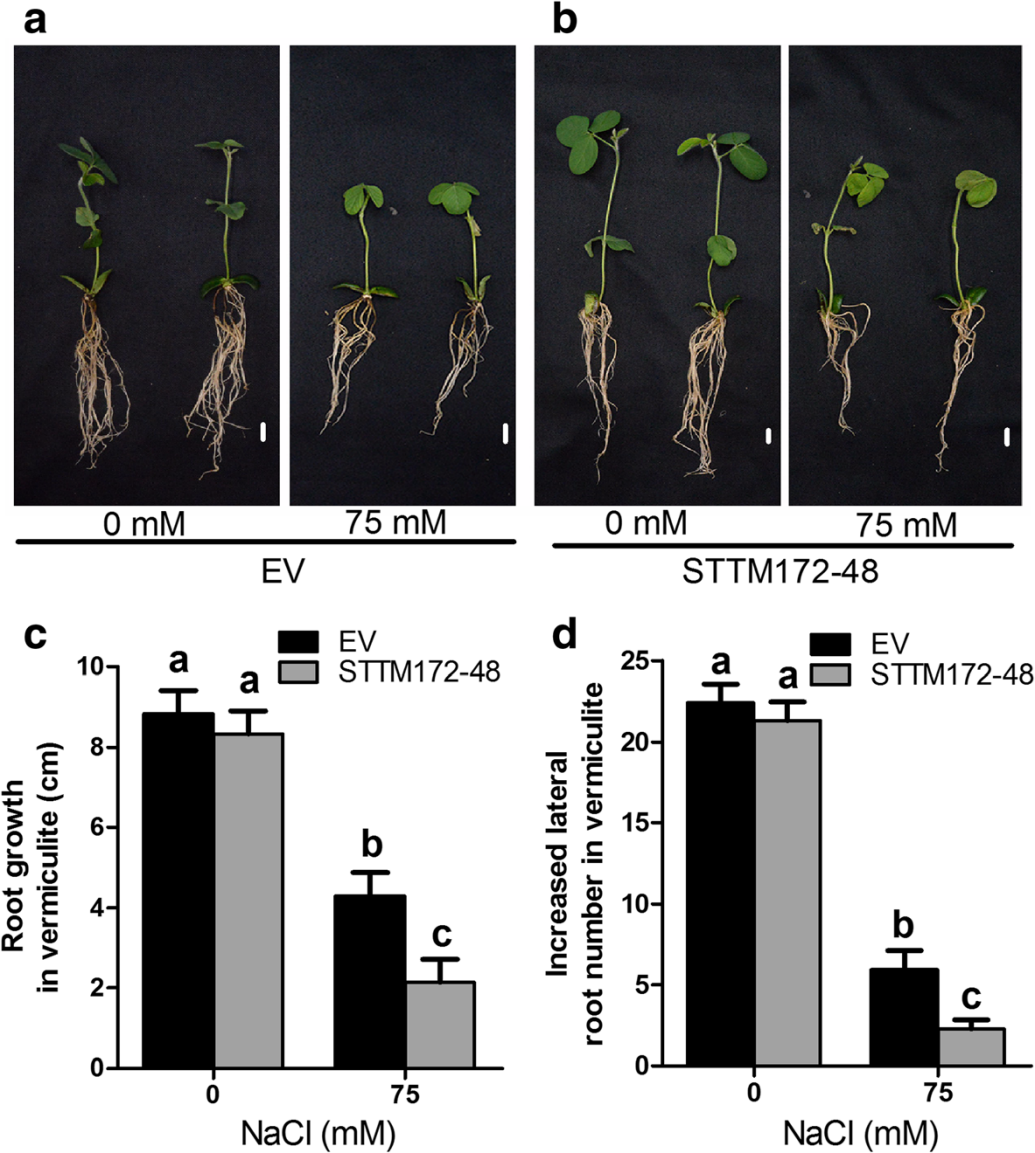
Reduction in miR172c activity increases salt sensitivity of soybean roots. **a**, **b** Phenotype analysis of composite plants and individual hairy root overexpressing the empty vector and STTM172–48. Seven days-old composite plants grown in B5 medium were transplanted to vermiculite and treated without (**a**) or with75 mM NaCl (**b**). Bars = 1 cm. **c**, **d** Root length (**c**) or lateral root number (**d**) of the composite plants expressing the empty vector and STTM172–48 after treatment Letters indicate significant differences from the empty vector controls according to the Student’s Newman-Kuels test (*P* < 0.05). Data shown above are all with three biological replicates.

To further validate the role of miR172c in root growth and plastic development under salt stress, we performed the same experiment with the composite transgenic plants with reduced activity of miR172 by expressing an STTM172–48 composed of two short sequences mimicking the miR172c target site separated by a linker as described previously [27]. miR172c expression was successfully reduced in the transgenic hairy roots expressing STTM172–48 (Additional file 2). Similarly, in the absence of salt, root system growth of the STTM172–48 composite plants and the vector controls at 15 days after transplanting to soil were comparable (Fig. 3). By contrast, the root system of the plants expressing STTM172–48 exhibited increased sensitivity to salt stress compared with that of control (Fig. 3a, b). At 15 days after salt treatment in soil, the root growth was greatly retarded, and lateral root development was dramatically inhibited (Fig. 3c, d). Together, these results suggest that the endogenous miR172c gene has a specific role in soybean root plastic development under salt stress.

### The target gene *NNC1* of miR172c is responsive to salt stress

**Fig. 4:**
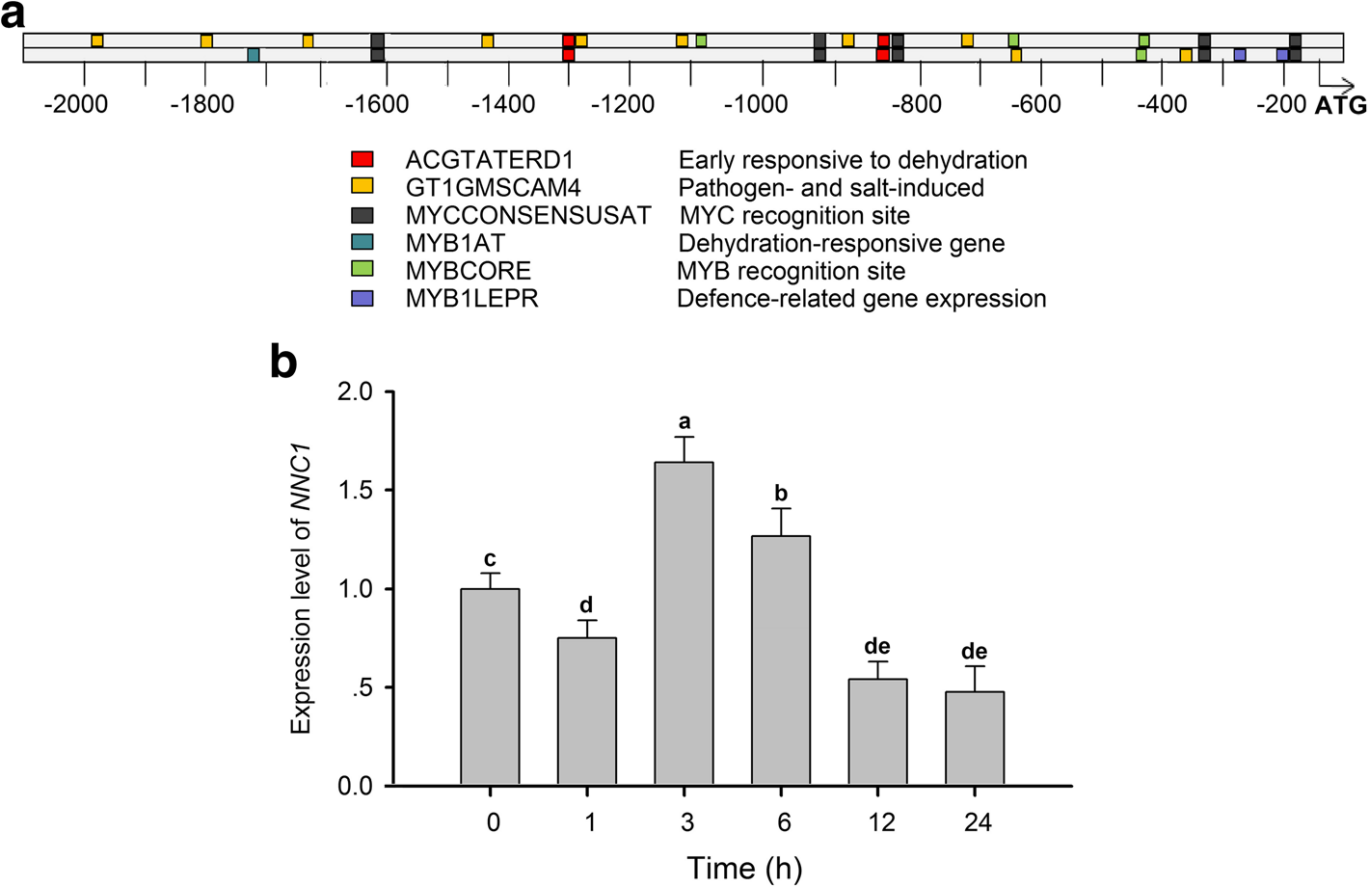
Analysis of *NNC1* promoter and expression under salt stress. **a** Computation analysis of the *NNC1* promoter. Promoter sequence (2000 bp) of upstream of the CDS of *NNC1* (www.phytozome.net/) was chosen for multiple *cis*-element analysis using the online software (http://www.dna.affrc.go.jp/place/). **b** Relative expression of *NNC1* in roots in response to salt stress. Seven day-old seedlings were treated with 75 mM NaCl, and roots were harvested at 0, 1, 3, 6, 12, and 24 h after treatment. *GmELF1b* was used to normalize transcript abundances. Letters indicate significant differences from the empty vector controls according to the Student’s Newman-Kuels test (*P* < 0.05)

Previously we have demonstrated that soybean *NNC1* gene is a functional target gene of miR172c in nodulation [27]. To see whether the *NNC1* gene also mediates the miR172c-based root plastic development under salt stress, we first performed the promoter analysis to see whether there is a connection between *NNC1* and stress response. Interestingly, we found that the promoter of *NNC1* also contains multiple *cis*-regulatory elements which are analogous to those identified in miR172c (Fig. 1a; Fig. 4a). The results indicated that *NNC1* may also be responsive to salt stress and it could be a downstream gene that is directly targeted by the miRN172c in plant response to salt stress.

To verify the prediction, we first performed qPCR analysis to check whether NNC1 transcription is affected by salt stress. The result showed that *NNC1* gene was indeed responsive to salt treatment (Fig. 4b). The overall expression level of *NNC1* was down-regulated when the plants were exposed to prolonged salt stress. The pattern of *NNC1* expression was in sharp contrast to miR172c, which showed continuous up-regulation within a period of 24 h treatment with salt stress (Fig. 1b). The nearly opposite patterns of *NNC1* and miR172c in response to salt stress suggest that *NNC1* is a possible target for miR172c under salt stress in soybean. To validate *NNC1* is the main target of miR172c in response to salt stress, we further analyzed the expression of 6 putative target genes including *NNC1* [27, Additional file 3] in roots of the miR172c overexpressors and the vector controls treated with and without salt stress. The results showed that among the putative target genes, the levels of NNC1 gene were the lowest in the miR172c overexpression roots regardless of salt stress (Additional file 3), suggesting that *NNC1* is a main target of miR172c in salt tolerance. In addition, expression of *glyma.01 g188400* was greatly downregulated in salt-treated miR172c overexpressors compared with that of the untreated miR172c composite roots. The results indicate that miR172c may modulate root responses to salt stress through multiple targets.

### Knockdown of *NNC1* increases salt tolerance of roots

**Fig. 5:**
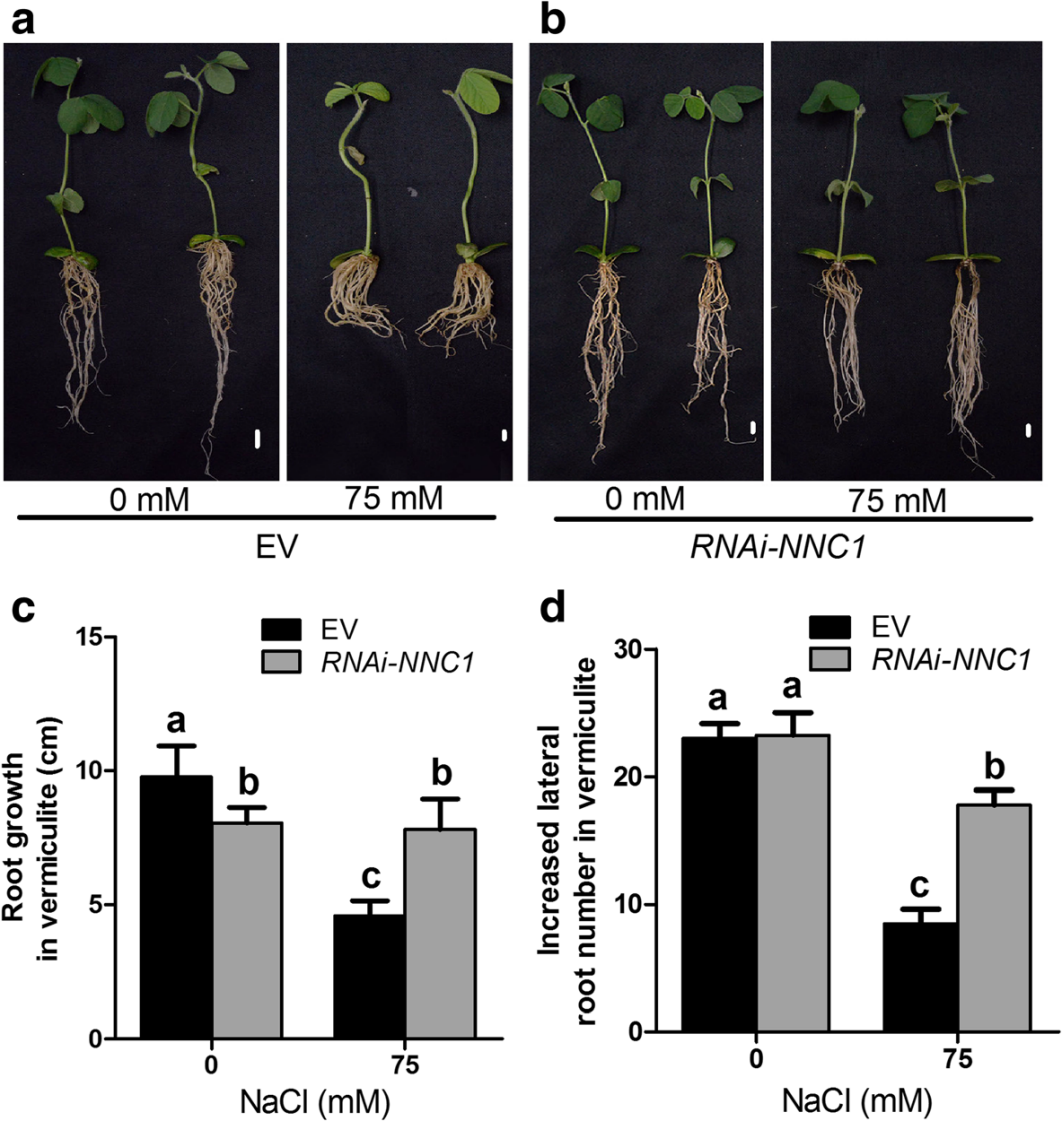
Reduction of *NNC1* expression increases salt tolerance of soybean. **a**, **b** Phenotypes of composite plants and individual hairy root expressing the empty vector and RNAi-*NNC1*. Seven days-old composite plants were transplanted to soil treated without (**a**) or with 75 mM NaCl. Bars = 1 cm. **c**, **d** Hairy root length and lateral root number of RNAi-*NNC1* under salt stress. **c**) Primary hairy root length of fifteen days old composite plant expressing the empty vector and RNAi-*NNC1*, **d**) Later root number of fifteen days old transgenetic hairy root expressing the empty vector and RNAi-*NNC1* (*n* = 10). Letters indicate significant differences from the empty vector controls according to the Student’s Newman-Kuels test (*P* < 0.05)

If the *NNC1* gene is the main target of miR172c in root salt response, knockdown of the gene would produce the similar phenotypes to the miR172c overexpression lines, i.e. enhanced tolerance of root to salt. To examine whether it is the case, we generated the composite transgenic plants with reduced expression of *NNC1* using RNA interference (*RNAi-NNC1*) and treated plants as described above (Additional file 4a, c). During hairy root initiation stage, *RNAi-NNC1* root system displayed stronger growth in both root elongation and lateral development than the vector control (Additional file 4b). After transplanting to soil without salt for 15 days, the root growth of *RNAi-NNC1* plants was unexpectedly slower than that of the vector control, but the average number of lateral roots formed per *RNAi-NNC1* hairy root after transplantation showed no significant difference with that per the control hairy root (Fig. 5a, b). In sharp contrast, the composite plants with the reduced expression of *NNC1* displayed substantially enhanced tolerance to salt stress compared with that of control (Fig. 5a, b). We found that both primary root growth and lateral root development of the *RNAi-NNC1* roots was greatly improved compared with that of the vector control at 15 days after salt stress (Fig. 5c and d). Apparently, *NNC1* is a negative regulator of root tolerance to salt stress in soybean. The opposite role of *NNC1* to miR172c in salt tolerance of soybean roots confirms that *NNC1* is a functional target of miR172c in plant response to salt in soybean.

### Over expression of the mutated *NNC1* increases salt sensitivity of soybean roots

**Fig. 6:**
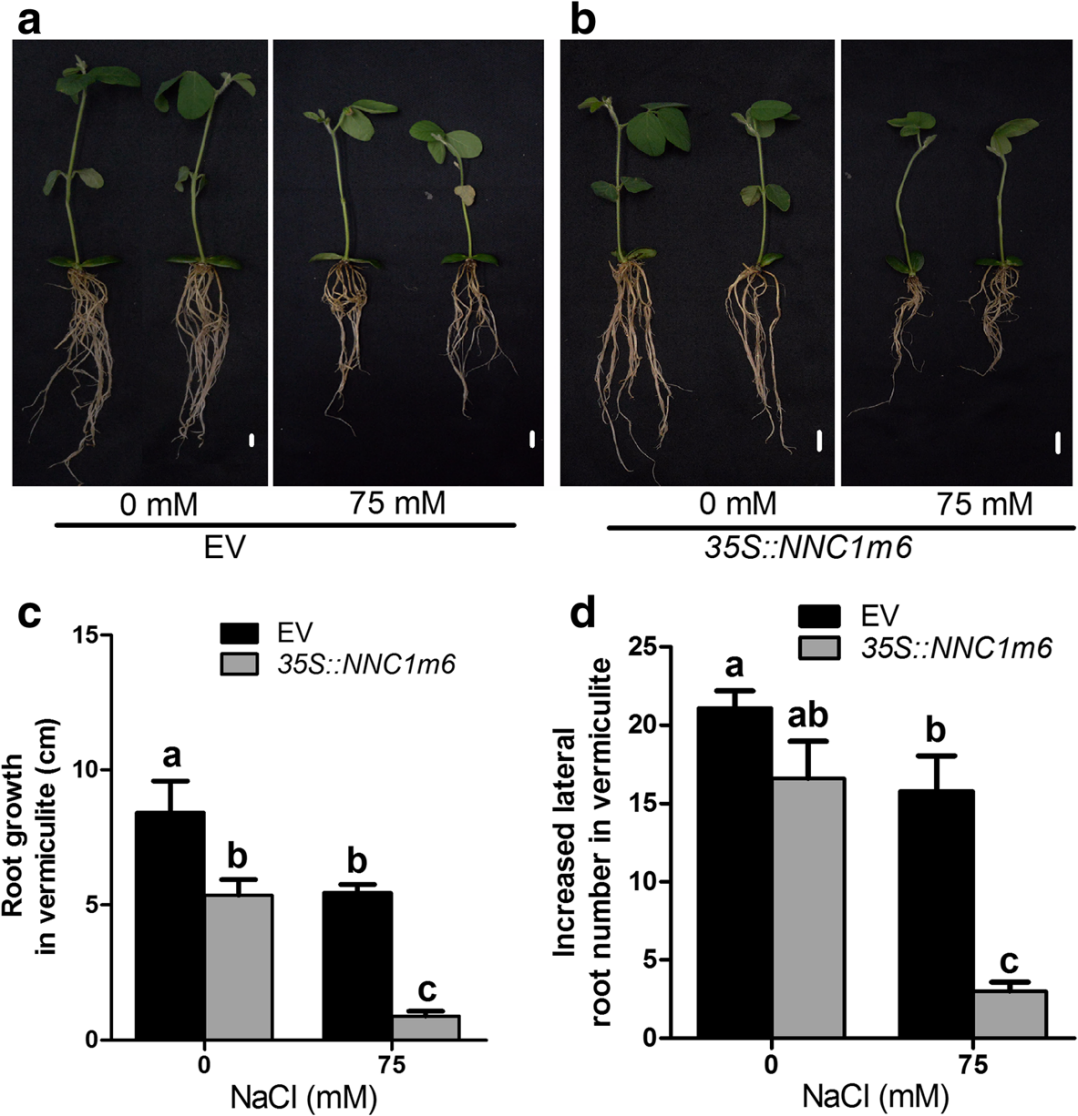
Overexpression of the mutated *NNC1* (*NNC1* m6) decreases salt resistance of soybean roots. **a**, **b** Phenotype of composite plants and individual hairy root expressing the empty vector and *NNC1*m6 under salt and control condition. Seven days-old composite plants grown in B5 medium were transplated to soil treated without (**a**) or with 75 mM NaCl (**b**). Bars = 1 cm. **c**, **d** Hairy root length (**c**) and lateral root number per hairy root (**d**) of the composite plants expressing the empty vector and *35S::NNC1*m6 at 15 days after treatment fifteen days old. (n = 10) Letters indicate significant differences from the empty vector controls according to the Student’s Newman-Kuels test (*P* < 0.05). Data shown above are all with three biological replicates.

To further validate whether *NNC1* is the main target of miR172c in the plastic development of root system under salt stress, we also over-expressed *NNC1* with six mismatches (*NNC1m6*) that cannot be recognized and cleaved by miR172c in transgenic roots [27]. As shown in Additional file 5c, *NNC1* transcription levels in the transgenic roots were markedly elevated compared with that in the vector control roots. Phenotypic analysis showed that over-expression of *NNC1m6* exhibited similar hairy root initiation rates to the vector control (Additional file 5a, b). However, when these *NNC1m6* overexpression composite plants grew in soil for another 15 days under normal conditions, root system growth during this period of time were markedly reduced (Fig. 6a and b). The length of transgenic roots and number of lateral roots per transgenic hairy root were substantially decreased compared to empty vector control (Fig. 6c, d). Notably, the roots of the composite plants over-expressing *NNC1m6* exhibited significantly increased sensitivity to salt stress when treated with 75 mM NaCl (Fig. 6). Root growth and newly formed lateral root per root of the *NNC1m6* composite plants were significantly reduced (Fig. 6c and d). The negative effect of *NNC1m6* over-expression on root development and plastic root development is similar to that of miR172c knockdown (Fig. 3). Together, these results confirmed that *NNC1* is a functional target of miR172c and act as a negative regulator in soybean root response to salt stress.

### Expression pattern of downstream genes are affected in transgenic *NNC1* roots under salt stress

**Fig. 7:**
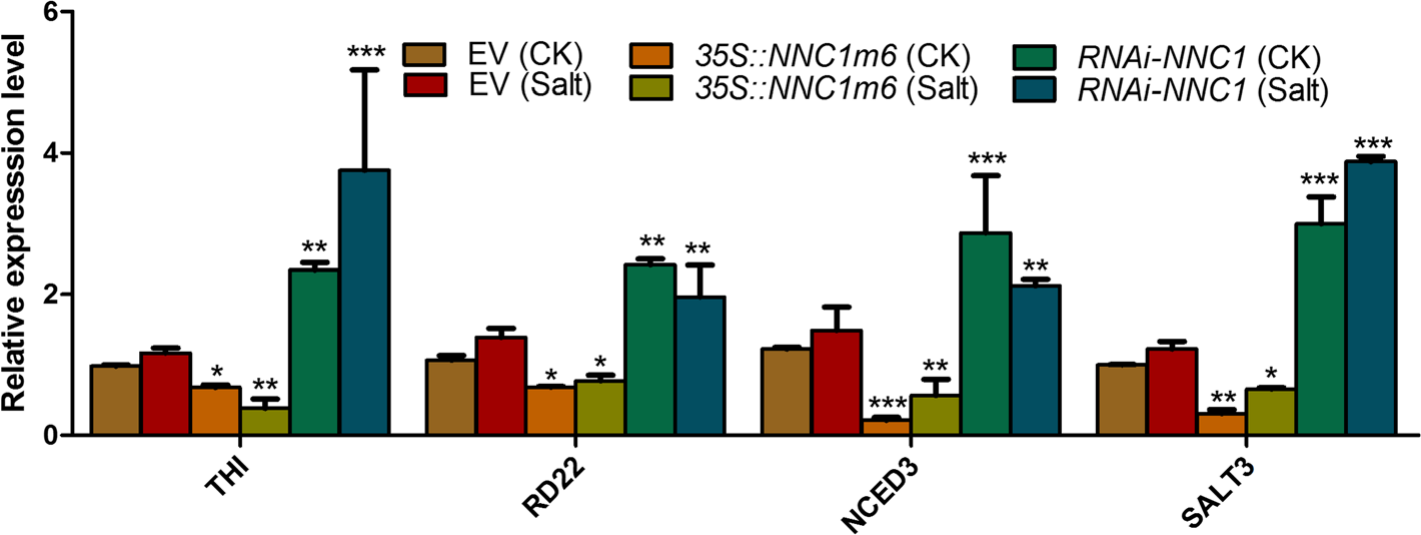
Relative transcript levels of *THI*, *RD22*, *NCED3* and *SALT3* in transgenic hairy roots. Seven day-old composite transgenic plants were treated with 75 mM NaCl and the root samples were harvested for RNA extraction. Relative transcript levels of *THI*, *RD22*, *NCED3* and *SALT3* in *35S::NNC1m6* and *RNAi-NNC1* transgenic roots compared to empty vector roots under normal and salt stress conditions. *GmELF1b* was used as the internal control. The data are the means ± SD of three replicates, and asterisks indicate a significant difference compared with the corresponding controls (*P < 0.05; **P < 0.01; ***P < 0.005)

To further gain insight into the mechanism of miR172c/*NNC1-*mediated plant salt responses, the expression levels of several downstream salt responsive genes, including *THI*, *RD22*, *NCED3* and *SALT3*, were analyzed in the transgenic roots of *35S::NNC1m6* and *RNAi-NNC1* and their empty vector control roots under normal and salt stress conditions [30]. The transcript levels of *THI*, *RD22*, *NCED3* and *SALT3* were significantly decreased in the *35S::NNC1m6* transgenic root compared with the vector control under both salt and normal conditions (Fig. 7); by contrast, the expression levels of all the tested genes, *THI*, *RD22*, *NCED3* and *SALT3*, were significantly higher in the *RNAi-NNC1* transgenic root compared with the vector control under both salt and normal conditions (Fig. 7). These results suggest that *NNC1* may modulate soybean root response to salt stress viathese downstream salt responsive genes.

## Discussion

As a sessile organism, plants constantly encounter changing environment including ion content in the soil. Therefore, it is critical for plants to make rapid regulatory reprogramming that remodels the cellular status and changes the cell fate to adapt the environment. Because of important regulatory roles of transcription factors, extensive studies focused on the transcriptome of transcription factors in response to salt stress decade ago [31]. Many transcription factors have identified to mediate plant salt tolerance [32, 33]. In the past several years, miRNAs have also been recognized as primary gene regulators [12, 34], and they likely function upstream of the transcription factors to perform their regulatory roles in plant salt tolerance based on computational prediction and experimental validation for direct interaction between miRNAs and transcription factors. Despite extensive global analysis of miRNAome data in various plants, the functional characterization of miRNAs and their transcription factors targets in the salt response of plants are still rare. In the current study, we showed convincing evidence that the miR172c-AP2 transcription factor NNC1 module is required for salt tolerance in soybean’s roots.

miR172 is a conserved miRNA family in plants [35, 36], however, the numbers of miR172 family members vary remarkably [27]. In Arabidopsis, miR172 family has two members, and both of them are key regulators in developmental phase transition from vegetative growth to reproductive development [24, 36]. However, our previous results also showed that Arabidopsis miR172b also mediates plant response to abiotic stress through targeting *SNZ* via an ABA-dependent pathway during early development [37]. Apparently, we can conclude that miR172 not only mediates plant development but also plant response to abiotic stress. The global analyses of miRNAs in plants treated with salt stresses favor the hypothesis because miR172 family members have been identified as the salt-responsive genes although the members of miR172 family genes behave differently in various plant species or different tissues of a plant species [19, 37–39]. For example, miR172a was highly responsive to salt in radish (*Raphanus sativus* L.) [16], whereas miR172b was the one family member that may play more important role in plant response to salt stress [39, 40]. Here, we revealed miR172c as a key regulator of plant salt tolerance in soybean roots. We showed that miR172c was highly responsive to salt treatment in roots (Fig. 1). In the presence of salt stress, ubiquitous expression of miR172c in soybean roots indicates that in addition to lateral root development, miR172c may also be involved in more cellular responses to salt stress, including ion limitation, ion homeostasis and so on. Most importantly, we demonstrated that miR172c positively regulates root plasticity under salt stress (Fig. 2). Over-expression of miR172c significantly increases primary root growth and lateral root development of soybean under salt stress, whereas the reduction in miR17c activity increases root sensitivity to salt (Fig. 2 and Fig. 3). The important role of miR172c in plant stress tolerance was also proved by ecotopic expression of soybean miR172c in Arabidopsis [30, 41], highlighting soybean miR172c as potential genes for genetic engineering of plant tolerance to abiotic stress. Since there are 12 miR172 family members in soybean [27, 30, 41], we do not exclude the possibility that other family members may also mediate plant stress tolerance.

It is well known that miR172 functions through targeting the AP2/ERF transcription factor family genes [23]. Previously, we showed that there are 12 target genes of miR172 in soybean [27]. In this work, our results revealed that NNC1 is an important target gene in miR172c-mediated salt tolerance in soybean roots. Alteration in *NNC1* expression changes salt tolerance of roots to salt stress (Fig. 5 and Fig. 6). Previously, we showed that miR172c represses *NNC1* gene expression by direct cleaving of the NNC1 mRNA [27]. Therefore, we concluded that miR172c modulates plant root response and plastic development through the same molecular mechanism. In the most recent report, Li et al. showed that glyma1g39520, among the predicted 12 target genes, could be a direct target gene of soybean miR172c in stress response, and the role of the gene in plant stress response was proved ecotopic expression of the gene in Arabidopsis, another report also found that miR172a can function as a long distance signal to improve soybean salt tolerance [30, 41]. As miR172a and miR172c share the same candidate genes, so the redundancy of miR172a and miR172c in salt stress response is rational. These results implicated that miR172 may fine tune plant stress tolerance through a complex network involving different target genes that act cell/tissue specifically or spatially/temporally. Considering the expression pattern of miR172c in response to salt stress, it is likely that miR172c also reprograms physiological and metabolic pathways through different target genes. The previous results have shown that overexpression of soybean miR172c altered ROS contents and ABA sensitivity in Arabidopsis, supporting the above hypothesis. Further systematic and functional analysis of the miR172 and their target genes will enable us to fully understand the miR172-AP2/ERF target genes mediated regulatory mechanisms in plant development and response to abiotic/biotic stimuli including interaction with symbiotic rhizobia.

The interaction between miR172 and its AP2/ERF target genes in modulating various biological processes has set an excellent example for a regulatory network of miRNA-based transcription factors in plants. Identification of the downstream target genes of these transcription factors will elucidate how miR172/target genes modulate various biological processes, such as maintenance of ROS and ion homeostasis, hormonal sensitivity (e.G. *aba* and auxin) and so on. In addition, analysis of the *cis-*regulatory elements in the miR172c promoter led the discovery of the novel role of miR172c in soybean. Based on the *cis*-elements, we proposed that miR172c induction may be regulated by ABA signaling pathway and other upstream transcription factors that involved in abiotic stress responses. Further identification of the upstream regulatory genes that regulate responsiveness of miR172c to developmental or environmental signals will help us to decipher the molecular mechanism underlying miR172c-mediated plant stress response and root plastic development in soybean. Since miR172c promoter contains *cis* elements responsive to other plant hormones, such as auxin and cytokinin [27], miR172c may function as a node that integrates intrinsic and extrinsic signals to modulate soybean growth and plastic development.

## Conclusions

Besides the important role of miR172 in plant flowering and legume nodulation, we also unveiled its positive role in soybean salt tolerance through its target gene NNC1. Both miR172c and *NNC1* responded differently to salt stress. Functional analysis revealed that miR172c confers root plasticity development and tolerance to salinity while *NNC1*confers susceptibility to salt stress. Further application of miR172c and *NNC1*on soybean may improve its growth and production in the field.

## Methods

### Plant growth conditions and hairy root transformation

Healthy and uniform soybean (*Glycine max* cv William 82) seeds were selected from seed stock and were sterilized with chlorine gas for 12–14 h. Sterilized seeds were germinated in B5 medium for 4 days under 16 h:8 h Light:dark conditions in a growth chamber at 25–26 °C. Soybean germinating seedlings were used for hairy root transformation according to the previously described methods (Kereszt et al., 2007; Jian et al., 2009). *Agrobacterium rhizogenes* strain K599 was used for all the hairy root transformation experiments.

### Salt treatment and root phenotyping of the composite transgenic plants

After co-cultivation, the explants were transferred to MS rooting medium with and without NaCl treatment. The concentration of NaCl was 75 mM based on our previous results. Formation and growth of hairy roots of the composite transgenic plants were observed during a 15 days inspection period. Lengths of the transgenic roots and lateral root number per transgenic root were counted at 15 days after grown in rooting medium with or without salt. The pictures of the representative roots were taken and the average length of the hairy roots and the average number of lateral roots per hairy root were calculated as the mean ± SD. All of the samples were collected and frozen in liquid nitrogen and kept under −80° for further analyses. Each treatment contained at least 10 explants with approximately 50 hairy roots, and each experiment was repeated at least three times.

### Histochemical analysis of miR172c expression

Composite plants expressing *promiR72c::GUS* were generated through *Agrobacterium rhizogenes*-mediated hairy root transformation system. Transformed composite seedlings were grown in MS rooting medium for 15 days and then treated with salt (75 mM NaCl) for 24 h and then the whole hairy root system was stained with GUS. The transgenic roots grown in MS medium without salt were also used for GUS assay. GUS staining was performed as described previously (Jefferson et al., 1987).

### DNA extraction and PCR analysis of the transgenic roots

Composite plants were generated through *Agrobacterium rhizogenes*-mediated hairy root transformation system. The composite plants were then transferred to MS medium with or without salt (75 mM NaCl). After 15 days samples were collected for DNA extraction. Root DNA was extracted as described by Doyle and Doyle (1987), and then assessed by the *bar* gene through PCR using the specific set of primers, the primers sequence of the *bar* gene is listed in Additional file 6: Table S1.

### RNA extraction and quantitative PCR analysis

Total RNA were extracted from transgenic hairy roots using Trizol reagent (Tiangen Biotech). The total RNA samples were then treated with gDNA remover (Tiangen) to clean contaminating genomic DNA. cDNA strand was synthesized from the RNA by the Fast Quant RT Kit (Tiangen Biotech). qRT-PCR was done using Super Real Pre-Mix Plus (SYBR Green; Tiangen Biotech) and gene-specific primers for the genes analysis are listed in Additional file 6: Table S1. *ELF1b* was used as the internal control (Jian et al., 2008).

### Stem-loop qRT-PCR

Stem-loop-specific reverse transcription for miR172c was performed as previously described (Chen et al., 2005; Kulcheski et al., 2010). Soybean miR1515a was used as an internal control for the miRNA gene to normalize the samples as outlined previously (Kulcheski et al., 2010). Mature sequences of miR172c were used to design primers according to Chen et al. (2005). All primers used for stem-loop qRT-PCR are listed in Additional file 6: Table S1.

### Vector construction

Vector constructions for *promiR172::GUS* in pTF102, *35S::miR172c* in pEGAD, STTM172–48 in pEGAD, *RNAi-NNC1* in pTCK303 and *35S:*:*NNC1* m6 pTF101-GFP were used in this study, the detailed information for preparation of the constructs can be found in our previous paper (Wang et al., 2014).

### Bioinformatics analysis of the promoters

The fragments situated 2000 bp upstream of pre-miR172c or the ATG of *NNC1* (http://www.phytozome.net) were chosen as the promoter sequences for miR172c and *NNC1*. The *cis-*regulatory elements in the promoter regions of miR172c and *NNC1* were analyzed using the online software PLACE (http://www.dna.affrc.go.jp/PLACE).

### Statistical analysis

Data analysis was done using SigmaPlot 10.0 (Systat Software). The averages and standard deviation (SD) of all results were calculated, For all the statistical results, means and SD were calculated. Student’s t test and ANOVA were applied to generate *P* values. Student-Newman-Kuels tests were conducted when statistically significant differences exist.

## Additional files

Additional file 1: Root emergence and expression analysis of overexpressing miR172c root (TIFF 298 kb)

Additional file 2: Root emergence and characterization of STTM172–48 expressing root (TIFF 500 kb)

Additional file 3: Expression analysis of putative target genes of miR172c in response to salt stress (TIFF 122 kb)

Additional file 4: Root emergence and expression analysis of RNAi-NNC1 expressing root (TIFF 334 kb)

Additional file 5: Root emergence and expression analysis of 35S::NNC1m6 expressing root (TIFF 351 kb)

Additional file 6: Table S1. Primers used in this study (XLSX 10 kb)

## Abbreviations

ABA: Abscisic acid
ABRE: ABA response element
AP2: Apetala 2
bZIP: Basic leucine zipper
DREB: Dehydration responsive element binding protein
ERF: Ethylene response factor
GmSALT3: Salt tolerance-associated gene on chromosome 3
NAC: NAM, ATAF1/2, and CUC2
NNC1: Nodule number control
ROS: Reactive oxygen species
SNZ: SCHNARCHZAPFEN
STTM: Short tandem target mimic
