# Supplementary figures and images for: The miR172c-NNC1 module modulates root plastic development in response to salt in soybean
Source: BMC Plant Biol. 2017 Dec 1;17:229. doi: 10.1186/s12870-017-1161-9 (PMC5709930; doi:10.1186/s12870-017-1161-9)

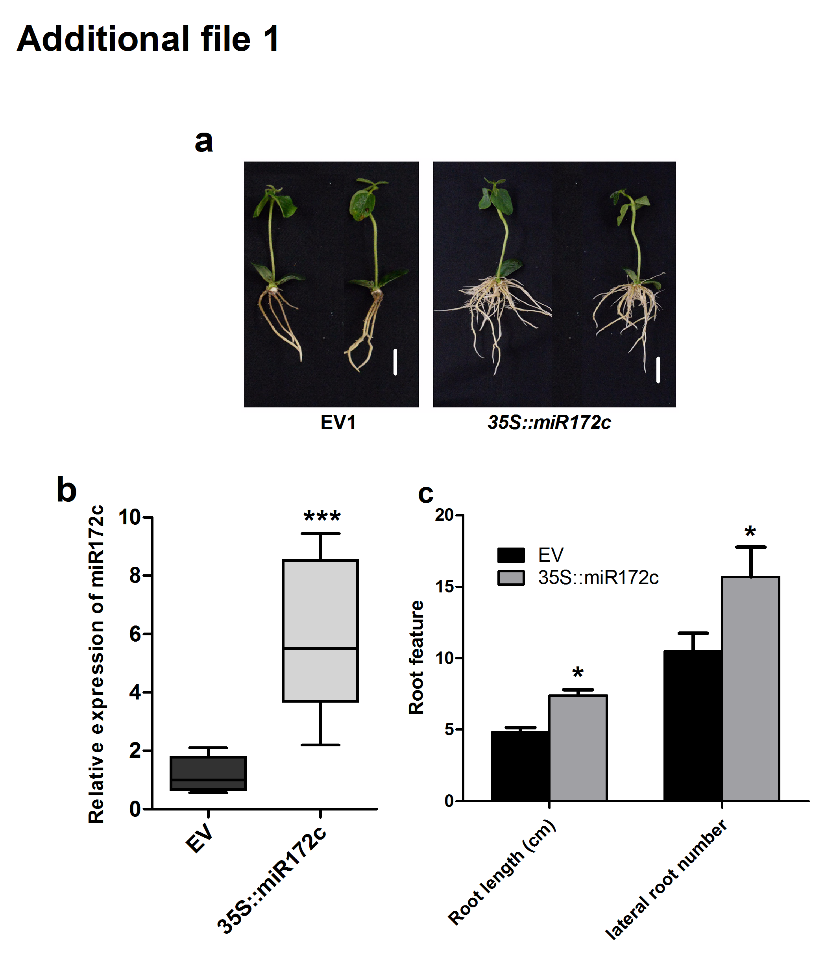

Supplement: Supplementary file 1 — Root emergence and expression analysis of overexpressing miR172c root (TIFF 298 kb) [file 12870_2017_1161_MOESM1_ESM.tif]

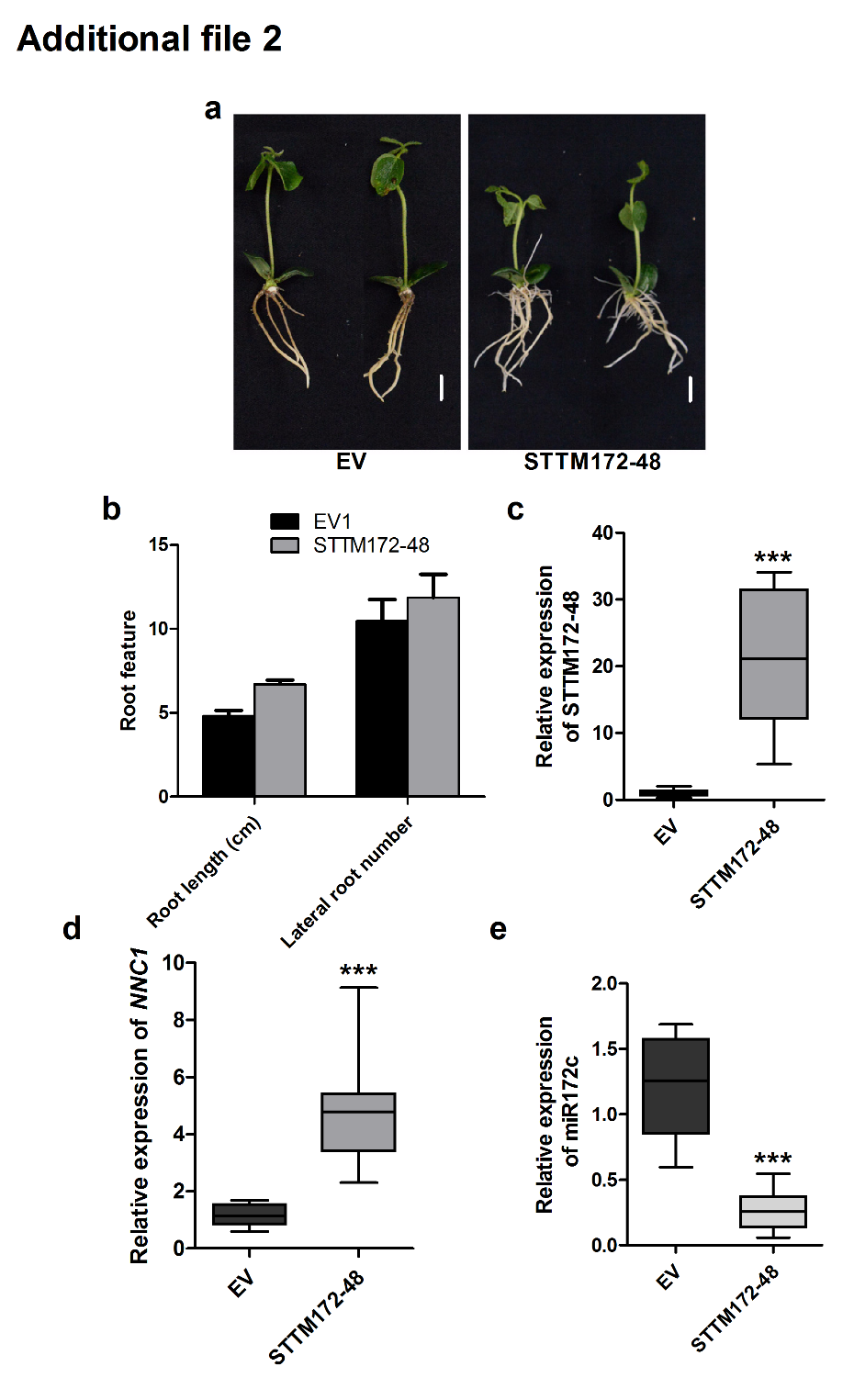

Supplement: Supplementary file 2 — Root emergence and characterization of STTM172–48 expressing root (TIFF 500 kb) [file 12870_2017_1161_MOESM2_ESM.tif]

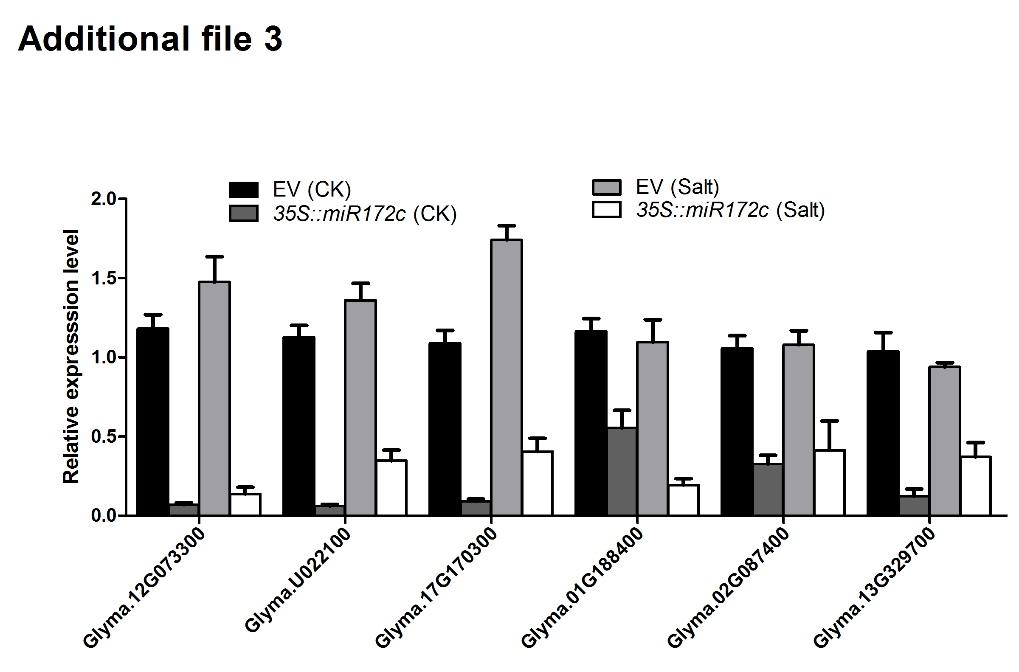

Supplement: Supplementary file 3 — Expression analysis of putative target genes of miR172c in response to salt stress (TIFF 122 kb) [file 12870_2017_1161_MOESM3_ESM.tif]

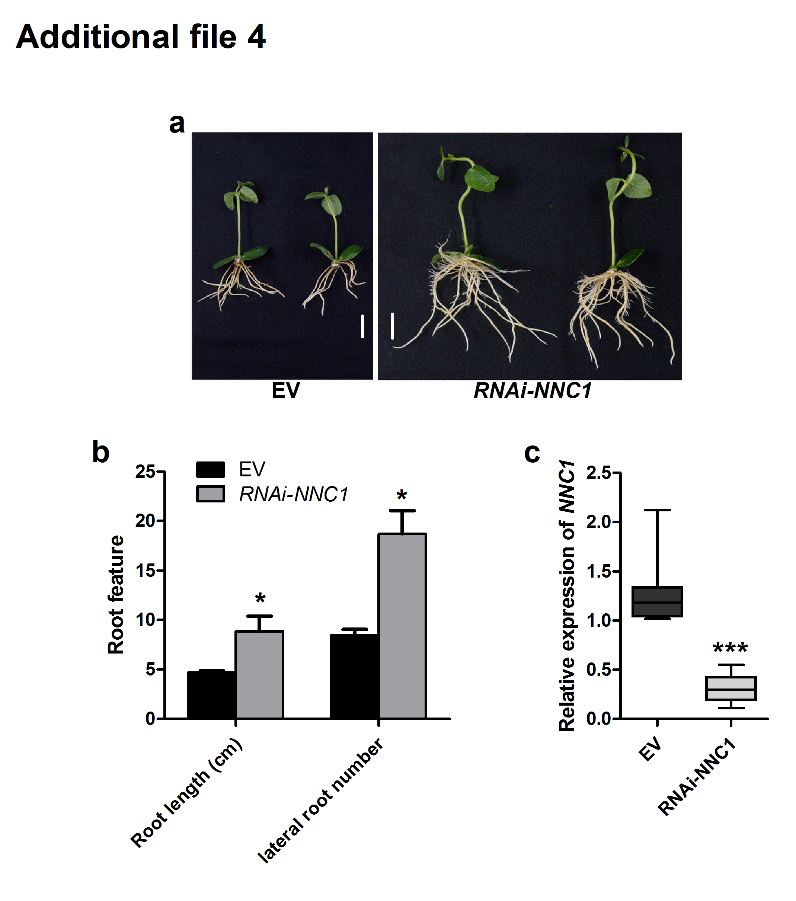

Supplement: Supplementary file 4 — Root emergence and expression analysis of RNAi-NNC1 expressing root (TIFF 334 kb) [file 12870_2017_1161_MOESM4_ESM.tif]

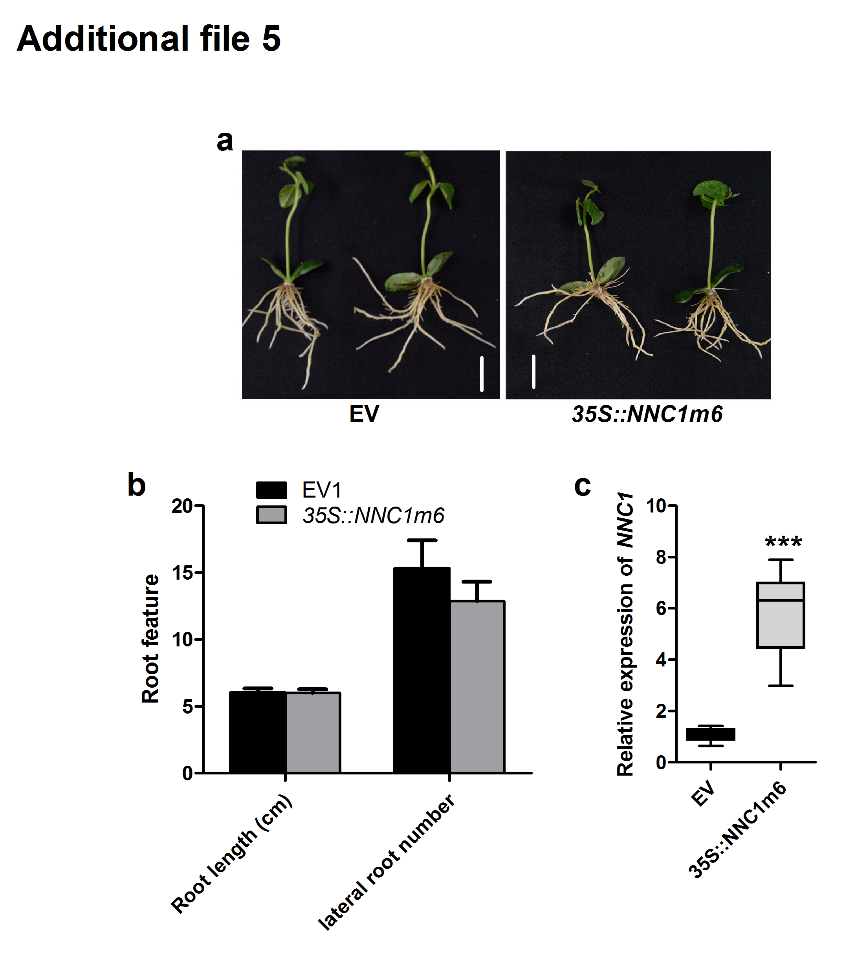

Supplement: Supplementary file 5 — Root emergence and expression analysis of 35S::NNC1m6 expressing root (TIFF 351 kb) [file 12870_2017_1161_MOESM5_ESM.tif]
